# Mesenchymal Stem Cell‐Derived Extracellular Vesicles Ameliorate NaIO_3_‐Induced Dry AMD by Delivering miR‐486‐3p to Inhibit Lipocalin‐2‐Mediated Ferroptosis

**DOI:** 10.1002/mco2.71000

**Published:** 2026-09-16

**Authors:** Zheng Li, Tujing Zhao, Lin Ye, Runze Li, Huaping Tian, Ruilin Liao, Lulin Huang

**Affiliations:** ^1^ The Genetic Diseases Key Laboratory of Sichuan Province The Department of Medical Genetics The Department of Laboratory Medicine Sichuan Academy of Medical Sciences & Sichuan Provincial People's Hospital School of Medicine, University of Electronic Science and Technology of China Chengdu 610072 China; ^2^ Chongqing Key Laboratory of Innovative Chinese Medicine and Health Intervention Chongqing Academy of Chinese Materia Medica Chongqing University of Chinese Medicine Chongqing 402760 China

**Keywords:** dry age‐related macular degeneration, extracellular vesicles, ferroptosis, Lipocalin‐2, miR‐486‐3p, retinal pigment epithelium

## Abstract

Dry age‐related macular degeneration (AMD), affecting over 196 million people globally, represents the leading cause of irreversible blindness with limited disease‐modifying therapies. The disease is characterized by progressive retinal pigment epithelium (RPE) degeneration driven by ferroptosis, an iron‐dependent form of regulated cell death. While mesenchymal stem cell (MSC)‐derived extracellular vesicles (EVs) show therapeutic promise, their mechanisms in counteracting RPE ferroptosis remain unexplored. Here, we prove that human umbilical cord MSC‐derived EVs (hucMSC‐EVs) significantly attenuate NaIO_3_‐induced retinal degeneration, preserving retinal structure and improving visual function in mice. Transcriptomic profiling identified Lipocalin‐2 (*Lcn2*) as a key ferroptosis‐related target. MSC‐EVs administration markedly downregulated Lcn2 and upregulated Gpx4 in NaIO_3_‐induced models, demonstrating potent ferroptosis suppression. Furthermore, AAV‐mediated *Lcn2* overexpression induced AMD‐like retinal pathology, which was effectively attenuated by subsequent MSC‐EVs treatment. Small RNA sequencing reveals miR‐486‐3p as the key factor in the MSC‐EVs, and dual‐luciferase reporter assays confirm its direct binding to the *Lcn2* 3'UTR. Functional validation demonstrates that miR‐486‐3p agomir recapitulates the effects of MSC‐EVs, including preservation of retinal structure and improvement of electrophysiological responses. Our findings establish a novel strategy where hucMSC‐EVs deliver miR‐486‐3p to suppress Lcn2‐mediated ferroptosis, offering a potential treatment for dry AMD.

## Graphical abstract

Our study reveals a novel mechanism by which MSC‐derived EVs alleviate NaIO_3_‐induced dry AMD‐like pathology. MSC‐EVs deliver miR‐486‐3p into retinal cells to directly inhibit the ferroptosis‐promoting target gene *Lcn2*. Concurrently, downregulated Lcn2 restores intracellular Gpx4 expression, thereby suppressing ferroptosis and rescuing retinal degeneration in AMD‐like model mice.

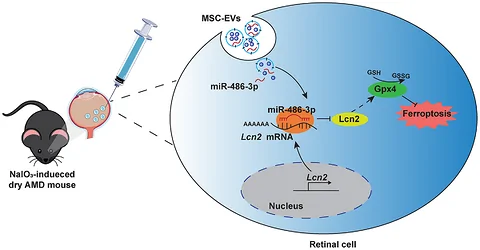

## 1 Introduction

Age‐related macular degeneration (AMD) represents a leading cause of irreversible blindness in the elderly population globally, affecting approximately 196 million people, with projections suggesting this number will reach 288 million by 2040, posing a significant public health challenge [1, 2]. Clinically, AMD manifests in two primary forms: neovascular (wet) AMD, accounting for 10%–15% of cases, and nonexudative (dry) AMD, representing 85%–90% of cases [3, 4]. While antivascular endothelial growth factor (anti‐VEGF) therapies offer a treatment modality for wet AMD, they often necessitate lifelong, regular intravitreal injections and do not address the underlying pathology [5, 6]. Crucially, effective treatments for dry AMD, particularly its advanced stage characterized by geographic atrophy (GA), remain limited, highlighting a critical unmet clinical need [3, 5].

The pathogenesis of dry AMD is complex, involving chronic oxidative stress, inflammation, and the accumulation of cytotoxic lipid byproducts within retinal pigment epithelium (RPE) cells. This progressive RPE dysfunction ultimately triggers photoreceptor degeneration and cell death within the macula, leading to vision loss [1, 4]. Emerging evidence implicates ferroptosis, an iron‐dependent, nonapoptotic form of regulated cell death characterized by overwhelming lipid peroxidation, as a pivotal driver of RPE demise in dry AMD [7, 8, 9, 10]. Ferroptosis is characterized by iron‐dependent accumulation of lipid peroxides, primarily due to glutathione peroxidase 4 (Gpx4) inactivation and subsequent glutathione (GSH) depletion. This process leads to overwhelming lipid peroxidation, particularly in the presence of labile iron pools, culminating in oxidative damage and regulated cell death [11]. The RPE, with its high metabolic activity, high iron content, and constant exposure to light‐induced oxidative stress, is particularly susceptible to ferroptosis, making this pathway an attractive therapeutic target for dry AMD intervention.

Extracellular vesicles (EVs), particularly exosomes (30–150 nm), have emerged as promising therapeutic vehicles due to their natural cargo‐delivery capabilities, low immunogenicity, and ability to cross biological barriers [12]. EVs derived from mesenchymal stem cells (MSCs), in particular, are known to harbor a rich cargo of proteins, lipids, and nucleic acids (including microRNAs), mediating potent anti‐inflammatory, immunomodulatory, and tissue‐protective effects [12, 13]. MSC‐EVs have demonstrated therapeutic efficacy in diverse disease models, including liver injury and nonalcoholic steatohepatitis [14, 15, 16, 17]. In ocular diseases, preliminary studies suggest MSC‐EVs can protect RPE cells from oxidative stress via Nrf2 signaling activation [18], modulate inflammatory responses in dry eye models [19], and promote retinal ganglion cell survival in glaucoma models [20]. However, the specific mechanisms by which MSC‐EVs counteract ferroptosis in dry AMD, the identity of key therapeutic cargo molecules, and their precise molecular targets remain largely unexplored. This knowledge gap represents a significant barrier to the development of optimized MSC‐EVs‐based therapies for ocular diseases.

Here, we investigate the therapeutic potential of human umbilical cord MSC‐derived EVs (hucMSC‐EVs) in a sodium iodate (NaIO_3_)‐induced mouse model that recapitulates key features of dry AMD pathogenesis, including RPE damage, photoreceptor loss, and visual function impairment. We demonstrate that hucMSC‐EVs, administered via either intravitreal injection or topical eye drops, significantly attenuate AMD‐like pathology and restore visual function through multiple complementary mechanisms. We identify Lipocalin‐2 (Lcn2), a protein implicated in iron homeostasis, inflammation, and RPE pathology, as a potential target by transcriptomics and establish that EV‐mediated suppression of Lcn2 inhibits ferroptosis in retinal cells.

Furthermore, we perform small RNA sequencing to identify miR‐486‐3p as a key effector in hucMSC‐EVs and demonstrate that it can directly target *Lcn2* mRNA to repress its function. Our findings establish a novel axis targeting the miR‐486‐3p/Lcn2/ferroptosis pathway, offering a mechanism‐based potential strategy for dry AMD treatment.

## 2 Results

### 2.1 Comprehensive Characterization of MSC‐Derived Extracellular Vesicles

To ensure the identity, purity, and functional quality of the extracellular vesicles (EVs) evaluated in this study, EVs were isolated from the conditioned medium of human umbilical cord‐derived mesenchymal stem cells (hucMSCs) using differential ultracentrifugation coupled with density gradient purification. Comprehensive characterization was performed according to the Minimal Information for Studies of Extracellular Vesicles (MISEV) 2018 guidelines and International Society for Extracellular Vesicles (ISEV) recommendations to establish rigorous quality control standards.

Nanoparticle tracking analysis (NTA) revealed a homogeneous population of vesicles with a size distribution characteristic of EVs, exhibiting a modal diameter of 152.4 nm and a concentration of 7.8 × 10^11^ particles/mL (Figure S1A). The size distribution was narrow (polydispersity index: 0.18 ± 0.03), indicating high homogeneity and consistent with high‐quality EV preparations. Transmission electron microscopy (TEM) visualization confirmed the presence of intact, membrane‐bound vesicles displaying the typical cup‐shaped morphology expected for EVs, with diameters ranging from 80–180 nm and well‐preserved bilayer membrane structure (Figure S1B). Western blot analysis was conducted to assess purity and identity. The EV preparations showed significant enrichment of canonical transmembrane (CD9, CD63) and intraluminal (TSG101) exosome markers (Figure S1C). Conversely, Calnexin, an endoplasmic reticulum protein commonly used as a negative control marker for cellular contamination, was abundant in cell lysates but undetectable in the purified EV fraction. Protein quantification revealed an EV yield of 1.5 µg/µL, with a protein‐to‐particle ratio of 5.2 × 10^8^ particles/µg protein, consistent with high‐quality EV preparations. These orthogonal characterization methods collectively validate the successful isolation of high‐quality hucMSC‐derived EVs, with minimal cellular contamination and suitable for subsequent functional studies. The characterization data meet or exceed the quality standards established by MISEV guidelines for EV research.

To further verify that hucMSC‐derived EVs can reach and distribute within the retina, we delivered PKH26‐labeled EVs via two routes: topical eye drops and intravitreal injection. Retinal flatmounts were examined to assess EV localization over time (Figure S2A–D). Following topical administration, red fluorescent EV signals were detected as punctate deposits by 3 h, progressively accumulated with peak intensity at 12 h, and slightly declined by 24 h (Figure S2A,B). In contrast, intravitreal injection resulted in rapid and robust EV accumulation throughout the retina within 1 day, with sustained high signal at day 3 and a marked reduction by day 6 (Figure S2C,D). To rule out potential dye‐related artifacts, an equivalent volume of PKH26 dye diluted in PBS was administered as a negative control (PKH26‐PBS).. Fluorescence imaging demonstrated that PKH26‐labeled EVs exhibited substantially higher accumulation in the retina compared with the PBS control group (Figure S2E,F). Cryosection analysis demonstrated that topically applied EVs successfully penetrated the ocular barriers, sequentially reaching the ganglion cell layer (GCL), inner nuclear layer (INL), and outer nuclear layer (ONL) within 12 h. Meanwhile, intravitreal injection conferred a widespread distribution of EVs throughout all retinal layers, with the fluorescence signal intensity progressively diminishing over a 6‐day observation period (Figure S2G,H). Additionally, immunofluorescence analysis demonstrated that hucMSC‐EVs localized beyond the neural retina to the outer retinal/RPE‐choroid complex, exhibiting clear co‐localization with the RPE‐specific marker RPE65 (Figure S2I). Furthermore, RPE flatmount analysis confirmed that PKH26‐labeled EVs accumulated significantly in the RPE layer compared with the PKH26‐PBS control group, via both topical administration and intravitreal injection (Figure S2J,K). Collectively, these findings demonstrate that hucMSC‐derived EVs can be delivered to the posterior retina through both noninvasive topical and intravitreal routes, supporting their potential application in the treatment of posterior segment eye diseases.

### 2.2 MSC‐Derived EVs Attenuate Retinal Degeneration and Rescue Visual Function in a Dry AMD Mouse Model

**FIGURE 1:**
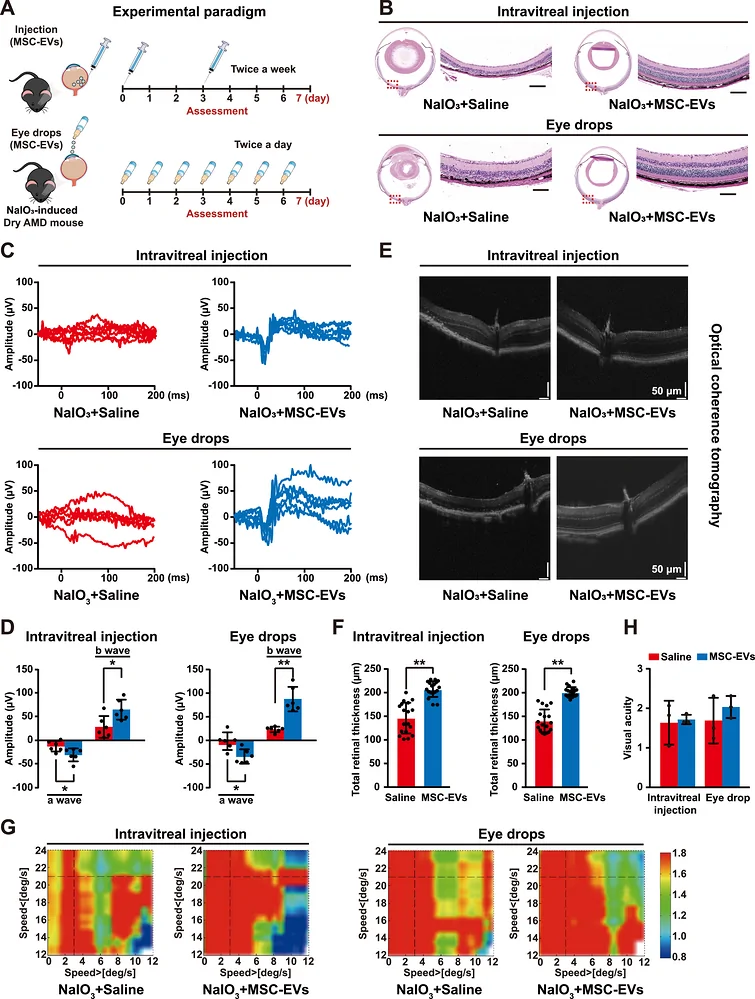
MSC‐EVs Attenuate NaIO_3_‐induced retinal degeneration and rescue visual function. (A) Experimental design for the MSC‐EVs administration for the NaIO_3_‐induced AMD‐like mouse model. The injected administration was performed on days 0 and 3, with a frequency of twice a week. The eye drops’ administration was performed twice a day during the 7‐day experiment. (B) Representative HE‐stained retinal sections from control, NaIO_3_+saline, NaIO_3_+MSC‐EVs (Intravitreal), and NaIO_3_+MSC‐EVs (eye drop) treated mice, showing preserved retinal structure with EVs treatment. Scale bar = 50 µm. (C) Representative scotopic ERG waveforms demonstrating improved retinal responses in MSC‐EVs‐treated groups. (D) Quantification of ERG a‐ and b‐wave amplitudes showing significant functional rescue by both intravitreal and eye drop MSC‐EVs administration. *n* = 3. (E) Representative OCT images illustrating improved retinal layer delineation and thickness in MSC‐EVs‐treated mice. (F) Quantification of retinal thickness from OCT images showing significant preservation with MSC‐EVs treatment. *n* = 3. (G‐H) OMR analysis indicating a trend toward improved visual acuity in MSC‐EVs‐treated groups. *n* = 3. Data are presented as mean ± SD. For significance: * *p < *0.05 and ** *p < *0.01 versus the control.

Having confirmed the quality of our hucMSC‐EVs preparations, we next investigated their therapeutic potential in the sodium iodate (NaIO_3_)‐induced AMD‐like mouse model, which recapitulates key features of RPE damage and subsequent photoreceptor loss observed in dry AMD [19, 21]. Following systemic NaIO_3_ administration (15 mg/kg, i.p.) to induce retinal pathology, mice received hucMSC‐EVs via either intravitreal injection or topical eye drops. The experimental design is shown in Figure 1A. In brief, for the injection administration, MSC‐EVs were injected on days 0 and 3, with a frequency of twice a week during the 7‐day experimental duration. Regarding the eye drops administration, MSC‐EVs eye drops were administered twice a day throughout the experimental period (Figure 1A). Control NaIO_3_‐treated mice received equivalent volumes of saline.

Histological examination by hematoxylin–eosin (HE) staining revealed severe disruption of retinal architecture in saline‐treated AMD‐like mice, characterized by significant thinning of the outer nuclear layer (ONL) (Figure 1B). In striking contrast, mice receiving hucMSC‐EVs, irrespective of the delivery route, exhibited markedly preserved retinal structure with ONL (Figure 1B). The preservation of retinal architecture was accompanied by reduced RPE vacuolization and improved cellular organization, as evidenced by reduced disorganization of retinal layers.

To assess the functional consequences of this structural protection, we performed electroretinography (ERG) under scotopic conditions. Systemic NaIO_3_ administration markedly suppressed scotopic a‐wave (indicative of photoreceptor function) and b‐wave (reflecting inner retinal function, predominantly bipolar cells) amplitudes (Figure 1C). Remarkably, intravitreal injection of MSC‐EVs achieved substantial functional rescue, elevating a‐wave amplitude from −14.14 ± 4.622 µV in the vehicle control group to −30.28 ± 5.77 µV, and b‐wave amplitude from −3.521 ± 1.439 µV to 64.92 ± 8.728 µV. Notably, topical administration of MSC‐EVs eye drops exhibited a comparable restorative effect (Figure 1D). This ERG recovery was consistently observed across varying flash intensities, underscoring the robust functional improvement imparted by MSC‐EVs treatment.

This functional recovery was corroborated by in vivo retinal imaging using optical coherence tomography (OCT). Compared with control AMD‐like mice, which displayed disorganized retinal stratification and marked thinning (total thickness: 141.9 ± 28.1 µm, MSC‐EVs‐treated mice exhibited well‐preserved retinal architecture and significantly enhanced total retinal thickness (198.9 ± 10.1 µm for intravitreal injection; 205.3 ± 16.4 µm for eye drops; Figure 1E,F). Detailed OCT cross‐sectional analysis further revealed superior structural preservation across individual retinal layers, most prominently within the outer nuclear layer (ONL) and inner nuclear layer (INL).

Assessment of the optomotor response (OMR), a behavioral measure of visual acuity, indicated a mild improvement in MSC‐EVs‐treated groups compared with saline‐treated controls but no significant difference. OMR scores increased from around 1.67 in NaIO_3_+saline to 1.72 ± 0.69 in intravitreal MSC‐EVs‐treated mice, and 2.04 ± 1.11 in eye drop‐treated mice, yet no statistically significant difference was observed (Figure 1G,H). The OMR improvement was consistent across spatial frequencies and contrast levels, suggesting some broad‐spectrum visual function improvement.

Taken together, these structural, physiological, and behavioral data provide compelling evidence that hucMSC‐EVs exert potent therapeutic effects, mitigating retinal degeneration and restoring visual function in this AMD‐like model. The dual administration routes both proved effective, with intravitreal injection showing slightly superior efficacy, likely due to direct posterior segment delivery and higher bioavailability.

### 2.3 Transcriptomic Profiling Identifies Lcn2 Downregulation Following MSC‐EVs Treatment

**FIGURE 2:**
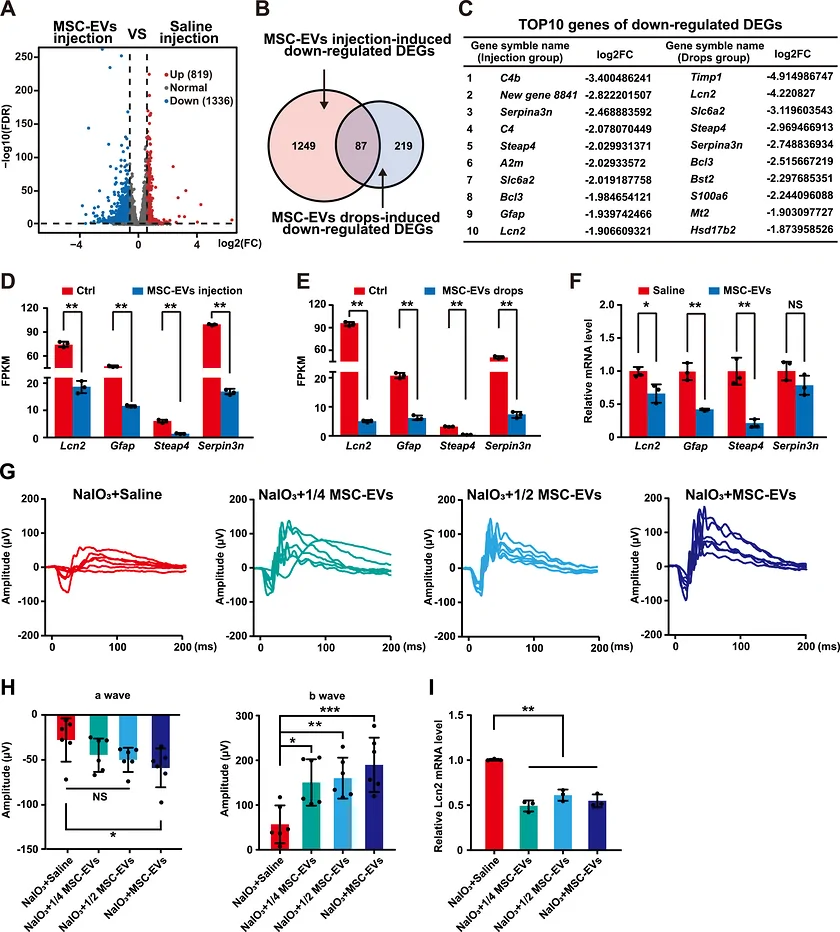
Transcriptomic profiling identifies *Lcn2* downregulation following MSC‐EVs treatment. (A) Volcano plot displaying differentially expressed genes (DEGs) in the retinas of NaIO_3_‐induced AMD‐like mice treated with MSC‐EVs via intravitreal injection versus saline controls. Red dots indicate significantly upregulated genes; blue dots indicate significantly downregulated genes. (B) Venn diagram illustrating the overlap of significantly downregulated DEGs identified following either intravitreal or eye drop EVs administration, highlighting 87 shared genes. (C) Table showing the relative expression levels of the top 10 shared downregulated genes across treatment groups. (D, E) RNA‐seq data of MSC‐EVs treated‐induced decrease in *Lcn2*, *Gfap*, *Steap4*, and *Serpin3n* mRNA expression in retina. FPKM, fragments per kilobase of transcript per million mapped fragments. (F) RT‐qPCR validation confirming significant downregulation of *Lcn2*, *Gfap*, and *Steap4* mRNA levels in the retinas of MSC‐EVs‐treated mice compared with saline controls. *n* = 6. (G) Representative scotopic ERG waveforms from NaIO_3_‐induced AMD‐like mice treated with different doses of MSC‐EVs by intravitreal injection. The 1/4 MSC‐EVs and 1/2 MSC‐EVs groups received one‐quarter and one‐half of the standard MSC‐EVs dose, respectively. (H) Quantification of ERG a‐ and b‐wave amplitudes in each group. *n* = 3. (I) Relative expression of *Lcn2* in the retina after treatment with different doses of MSC‐EVs. *n* = 3. Data are presented as mean ± SD. For significance: *p < 0.05 and **p < 0.01 versus the control.

To gain mechanistic insight into how hucMSC‐EVs mediate retinal protection, we performed RNA sequencing (RNA‐seq) on whole retinal tissues harvested from NaIO_3_‐induced AMD‐like mice treated with MSC‐EVs (via intravitreal injection or eye drops) or saline. Differential gene expression analysis revealed distinct transcriptomic signatures induced by MSC‐EVs treatment compared with saline controls (Figure 2A; Figures S3 and S4). Notably, intravitreal injection elicited a broader transcriptomic response (2155 DEGs) compared with eye drops (590 DEGs), potentially reflecting differences in bioavailability and target engagement (Figures S3 and S4).

Given that EVs often exert regulatory effects via transferred miRNAs leading to target mRNA suppression, we focused on genes significantly downregulated by both delivery routes. Intersection analysis identified a core set of 87 genes consistently downregulated by both intravitreal and topical EVs administration (Figure 2B). Gene Ontology (GO) analysis of these shared downregulated genes revealed enrichment in biological processes related to inflammatory response, immune system process, and response to oxidative stress, consistent with the anti‐inflammatory and antioxidative effects of MSC‐EVs.

Among the most significantly downregulated shared genes (Figure 2C), Lipocalin‐2 (Lcn2) emerged as a candidate of high interest, showing a significant reduction in expression. Lcn2 expression is known to be elevated in human AMD retinas [22, 23] and has recently been implicated in promoting RPE ferroptosis [24]. Pathway analysis revealed that Lcn2 is involved in iron homeostasis, inflammatory response, and cell death pathways, making it an attractive therapeutic target for AMD intervention.

RNA‐seq data showed that mRNA expression of *Lcn2* and other top candidates, including glial fibrillary acidic protein, Gfap (a marker of glial activation), STEAP family member 4 (Steap4), and serine (or cysteine) peptidase inhibitor, clade A, member 3N (Serpina3n), were dramatically decreased in the retinas of MSC‐EVs‐treated mice compared with saline‐treated controls (Figure 2D,E). We validated the RNA‐seq findings via Real‐Time Quantitative PCR (RT‐qPCR) on independent retinal samples. Results confirmed that, compared with controls, MSC‐EVs‐treated mice exhibited a significant downregulation of *Lcn2* mRNA (1.5 ± 0.1‐fold reduction), *Gfap* (2.3 ± 0.3‐fold reduction), *Steap4* (2.15 ± 1.9‐fold reduction), and *Serpina3n* (1.3 ± 0.2‐fold reduction) in the retinas (Figure 2F). The consistency between RNA‐seq and RT‐qPCR validation strongly supports the reliability of our transcriptomic analysis. We further used different concentrations of hucMSC‐EVs, via intravitreal injection, to treat the NaIO_3_‐induced AMD‐like mice. RT‐qPCR analysis showed that retinal *Lcn2* expression was significantly reduced in all EVs‐treated groups compared with the saline‐treated group. However, ERG analysis revealed that improvements in both a‐ and b‐wave amplitudes were observed only in mice receiving undiluted hucMSC‐EVs, whereas diluted EV preparations showed limited effects on retinal function recovery (Figure 2G–I). These results indicate that undiluted hucMSC‐EVs show a stronger suppression of *Lcn2* expression and the greatest effect in restoring retinal function.

### 2.4 MSC‐EVs Suppress Lcn2 and Inhibit Ferroptosis Markers in the AMD Model Retina

**FIGURE 3:**
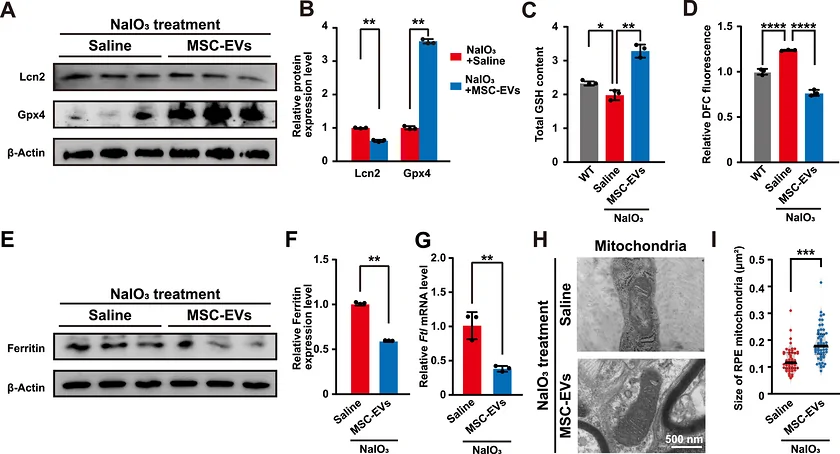
MSC‐EVs suppress Lcn2 and inhibit ferroptosis in the AMD‐like model retina. (A) Western blot analyses demonstrating significantly decreased Lcn2 and increased Gpx4 protein levels in the retinas of MSC‐EVs‐treated NaIO_3_‐induced mouse model. (B) Quantification of Lcn2 and Gpx4 protein expression in (A); *n* = 3. (C) Quantification of total glutathione (GSH) content revealing significantly elevated levels in the retinas of MSC‐EVs‐treated NaIO_3_‐induced mice; *n* = 3. (D) Relative DCF fluorescence level in different treatment group mice. (E–G) RT‐qPCR (G) and western blot (E) analyses demonstrating significantly reduced ferritin light chain (*Ftl*) mRNA and Ferritin protein expression in EVs‐treated NaIO_3_ mice; *n* = 3. (F) Quantification of Ferritin protein expression in (E). (H) Representative TEM images of RPE mitochondria from NaIO_3_+saline and NaIO_3_+ MSC‐EVs‐treated mice. Note the condensed, damaged mitochondria in the saline group versus the more preserved morphology in the MSC‐EVs‐treated group. Scale bar = 500 nm. (I) Quantification of mitochondrial area in RPE cells measured from TEM images; *n* = 3. Data are presented as mean ± SD. For the significance: * *p < *0.05, ** *p < *0.01, and *** *p < *0.001 versus the control.

Based on the observed reduction in Lcn2 expression and its previously reported role in ferroptosis [24], we reasoned that hucMSC‐EVs exert their protective effects against retinal damage by downregulating Lcn2, which subsequently attenuates the downstream ferroptosis signaling pathway. To test this hypothesis, we first confirmed Lcn2 suppression at the protein level in the retinas of MSC‐EVs‐treated AMD‐like mice via western blot (Figure 3A). Quantification results revealed a 1.7 ± 0.1‐fold reduction in Lcn2 protein levels (Figure 3B). Crucially, this reduction in Lcn2 expression following MSC‐EVs treatment was accompanied by a significant increase in the protein level by 3.6 ± 0.1‐fold of glutathione peroxidase 4 (Gpx4) (Figure 3A,B), the master enzyme responsible for detoxifying lipid peroxides and preventing ferroptosis [25]. We further examined key biochemical markers of ferroptosis to establish the functional consequences of Lcn2 suppression. Consistent with Gpx4 upregulation, the levels of its essential cofactor, glutathione (GSH), were significantly increased in the retinas of MSC‐EVs‐treated mice compared with controls (Figure 3C). Furthermore, excessive intracellular reactive oxygen species (ROS) accumulation was observed in retinal cells of AMD‐like mice, as evidenced by a marked elevation in DCF fluorescence intensity. Promisingly, treatment with MSC‐EVs effectively scavenged intracellular ROS and attenuated oxidative stress (Figure 3D).

Conversely, the expression of ferritin light chain 1 (*Ftl*), which encodes an iron storage protein Ferritin whose degradation can increase labile iron pools contributing to ferroptosis, was significantly reduced at both mRNA and protein levels following MSC‐EVs treatment (Figure 3E–G). *Ftl* mRNA expression decreased by 2.7 ± 0.5‐fold, while Ferritin protein levels decreased by around 1.7‐fold.

Finally, to assess the impact on cellular ultrastructure, we examined RPE cells using transmission electron microscopy (TEM). Transmission electron microscopy (TEM) revealed that RPE cells from saline‐treated AMD‐like mice exhibited classic ultrastructural hallmarks of ferroptosis, including compromised mitochondrial morphology characterized by condensed cristae, ruptured outer membranes, and extensive vacuolization (Figure 3H,I). Conversely, treatment with MSC‐EVs substantially preserved mitochondrial integrity, as evidenced by well‐defined cristae, intact outer membranes, and attenuated vacuolar degeneration (Figure 3H,I).

Collectively, these molecular and ultrastructural data provide strong evidence that hucMSC‐EVs treatment suppresses Lcn2 expression and concomitantly inhibits multiple key steps in the ferroptotic cell death pathway within the degenerating retina, including Gpx4 upregulation, GSH restoration, ferritin downregulation, and mitochondrial preservation.

### 2.5 Lcn2 Overexpression Induces Retinal Pathology, Which Is Rescued by MSC‐EVs

**FIGURE 4:**
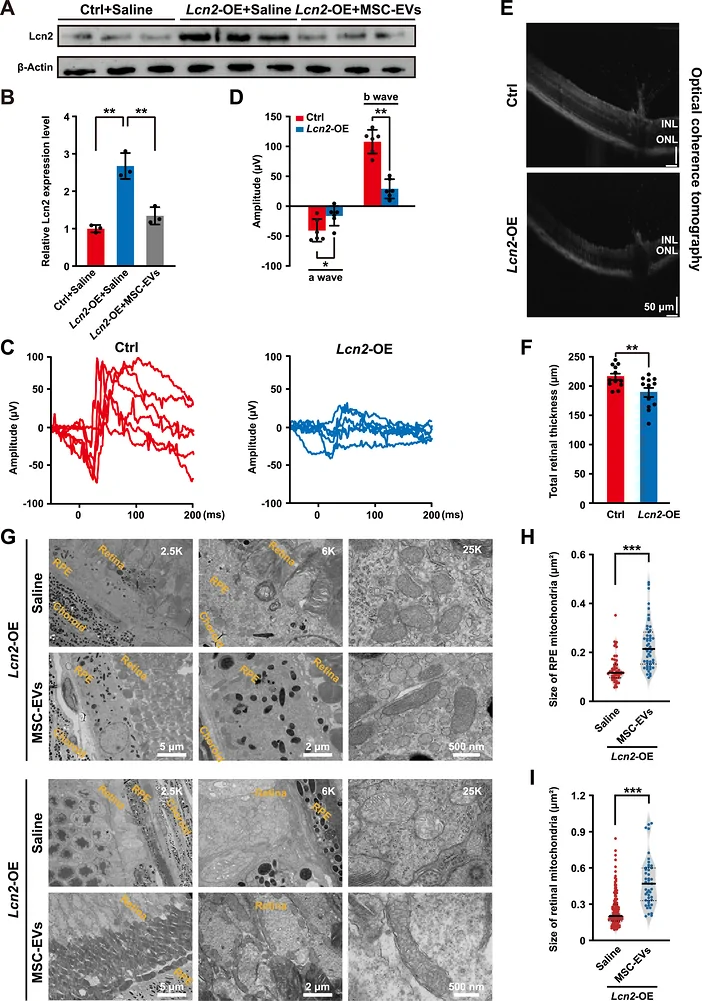
*Lcn2* overexpression induces retinal pathology, and this pathology is effectively rescued by MSC‐EVs. (A) Western blot analysis confirmed the successful overexpression of Lcn2 and its level decrease upon MSC‐EVs treatment. (B) Quantification of Lcn2 protein expression in (A). *n* = 3. (C) Representative scotopic ERG waveforms from *AAV‐Lcn2‐OE* injected mice, showing improved retinal responses by MSC‐EVs‐treated mice. Scale bar = 50 µm. (D) Quantification of ERG a‐ and b‐wave amplitudes demonstrating significant functional deficit in *AAV‐Lcn2‐OE* mice. *n* = 3. (E, F) Representative OCT images (E) and quantification of retinal thickness (F) showing retinal thinning and disruption caused by *Lcn2* overexpression. *n* = 3. (G) Representative TEM images of RPE/retina in *AAV‐Lcn2‐OE* mice treated with saline or MSC‐EVs. Note the disrupted structure and aberrant mitochondrial morphology in the saline group, which are improved following MSC‐EVs treatment. Scale bar = 5 µm (2.5K), 2 µm (6K), and 500 nm (25K). (H) Quantification of mitochondrial area in RPE cells measured from TEM images (6K). (I) Quantification of mitochondrial area in retinal cells measured from TEM images (6K); *n* = 3. Data are presented as mean ± SD. For significance: * *p < *0.05, ** *p < *0.01, and *** *p <* 0.001 versus the control.

To directly test whether elevated Lcn2 is sufficient to lead to AMD‐like pathology and to determine if EVs can counteract Lcn2‐mediated damage, we employed an adeno‐associated virus (AAV) vector to specifically overexpress *Lcn2* in the mouse retina (*AAV‐Lcn2*‐OE). Western blot analysis confirmed that intravitreal injection of *AAV‐Lcn2*‐OE (1 × 10^13^ viral genomes/mL) into wild‐type (WT) mice resulted in overexpression of Lcn2 protein levels (Figure 4A,B), which led to significant retinal pathology mimicking key aspects of the NaIO_3_ model within 14 days postinjection.

ERG recordings showed significantly attenuated a‐ and b‐wave amplitudes compared with control AAV‐injected mice (Figure 4C,D). OCT imaging confirmed retinal thinning and compromised layer integrity following *Lcn2* overexpression (Figure 4E,F), with total retinal thickness decreasing from 216.8 ± 18.7 µm in controls to 189.8 ± 27.2 µm in Lcn2‐overexpressing mice. RPE disruption was also evident, with increased vacuolization and cellular disorganization. These findings establish Lcn2 as a pathogenic factor capable of inducing retinal degeneration and functional impairment, providing a causal link between elevated Lcn2 expression and AMD‐like pathology.

We next investigated the role of hucMSC‐EVs in Lcn2‐ overexpressing mice. After receiving intravitreal injections of hucMSC‐EVs (2 µL/eye), the mice showed a substantial functional and structural rescue. TEM analysis demonstrated that MSC‐EVs treatment effectively restored the structural integrity of the RPE and retinal layers, which were disrupted by *Lcn2* overexpression (Figure 4G–I). Notably, the aberrant mitochondrial morphology (condensed cristae, swelling, membrane rupture) observed in RPE cells of *Lcn2*‐overexpressing mice was markedly improved following MSC‐EVs treatment, with mitochondria appearing more elongated and possessing well‐defined cristae (Figure 4G–I).

These results provide compelling evidence for a causal role of Lcn2 in retinal degeneration and demonstrate that hucMSC‐EVs can effectively protect against Lcn2‐mediated pathology, likely by both reducing Lcn2 levels and counteracting its downstream pro‐ferroptotic effects.

### 2.6 Exosomal miR‐486‐3p Mediates Therapeutic Effects by Targeting *Lcn2*

Given that MSC‐EVs primarily exert their bioactivity through the horizontal transfer of functional molecular cargo, coupled with our transcriptomic profiling highlighting Lcn2 downregulation, we reasoned that specific microRNAs (miRNAs) enriched within hucMSC‐EVs might mediate the posttranscriptional silencing of Lcn2.

**FIGURE 5:**
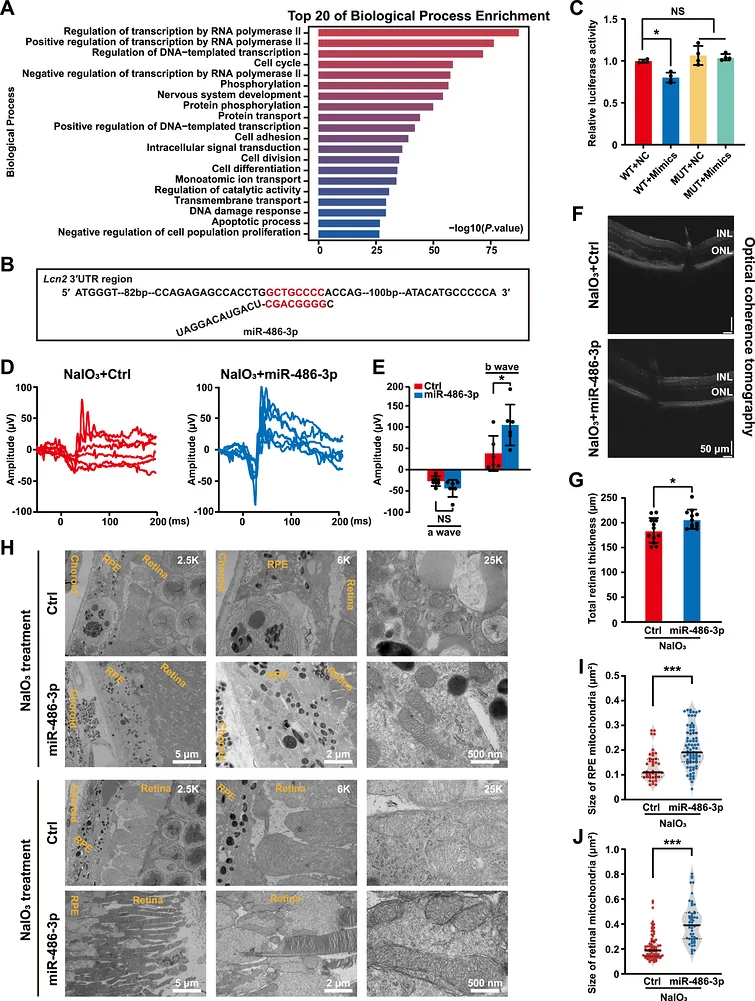
MSC‐EVs‐derived miR‐486‐3p mediates therapeutic effects by targeting *Lcn2*. (A) Gene Ontology (GO) analysis of predicted target genes for abundant miRNAs identified in MSC‐EVs by small RNA sequencing, highlighting enrichment in key biological processes. (B) Schematic representation of the predicted binding site for miR‐486‐3p within the 3'UTR of mouse *Lcn2* mRNA, based on mirDB prediction. (C) Dual‐luciferase reporter assay validating the interaction between miR‐486‐3p and the *Lcn2* 3'UTR. WT, wild‐type reporter construct; MUT, mutant reporter construct. *n* = 4. (D, E) Representative ERG waveforms (D) and quantification of a‐ and b‐wave amplitudes (E) showing significant functional rescue in NaIO_3_‐induced mice treated with miR‐486‐3p agomir compared with scramble controls. *n* = 3. (F, G) Representative OCT images (F) and quantification of retinal thickness (G) demonstrating structural rescue by miR‐486‐3p agomir. *n* = 3. (H) Representative TEM images showing restored RPE/retinal structure and improved mitochondrial morphology in NaIO_3_‐induced mice following miR‐486‐3p agomir treatment compared with scramble controls. Scale bar = 5 µm (2.5K), 2 µm (6K), and 500 nm (25K). (I) Quantification of mitochondrial area in RPE cells measured from TEM images (6K). (J) Quantification of mitochondrial area in retinal cells measured from TEM images (6K). *n* = 3. Data are presented as mean ± SD. For significance, **p < 0.01 versus the control.

Gene Ontology (GO) analysis of the predicted target genes for the most abundant miRNAs in the EVs implicated diverse biological processes, including transcriptional regulation, apoptosis, and iron homeostasis (Figure 5A; Table S1), highlighting the potential for broad regulatory impact. To identify potential Lcn2 regulators, we utilized mirDB bioinformatic prediction algorithms, which revealed a conserved binding site for miR‐486‐3p within the *Lcn2* 3' untranslated region (3'UTR) (Figure 5B; Table S2). The predicted binding site showed high conservation across mammalian species and a binding energy of −18.7 kcal/mol, indicating strong thermodynamic stability. Besides, the luciferase reporter assay results showed that miR‐486‐3p mimics significantly decreased the relative luciferase activity of the WT‐*Lcn2*‐3'UTR reporter construct. However, the mutant (MUT) of the predicted miR‐486‐3p binding site abolished this suppressive effect (Figure 5C). These results demonstrate that miR‐486‐3p specifically interacts with the *Lcn2* 3'UTR and suggest that *Lcn2* is a direct downstream target of miR‐486‐3p.

To functionally validate this prediction and determine if miR‐486‐3p alone could recapitulate the effects of the whole MSC‐EVs population, we administered synthetic miR‐486‐3p agomir (2 µg/eye) or scramble controls via intravitreal injection into NaIO_3_‐induced AMD‐like model mice. Strikingly, treatment with miR‐486‐3p agomir significantly rescued visual function, mirroring the effects of MSC‐EVs treatment. ERG recordings enhanced waveforms and significantly increased a‐ and b‐wave amplitudes (Figure 5D). Quantitative analysis revealed a trend toward increased a‐wave amplitude in the miR‐486‐3p agomir‐treated group compared with scramble‐treated controls (−26.15 ± 11.57 µV vs. −43.21 ± 20.13 µV), although the difference was not statistically significant. Notably, miR‐486‐3p agomir treatment significantly increased the b‐wave amplitude from 39.20 ± 41.48 µV to 106.2 ± 48.29 µV (Figure 5E). These improvements achieved 82.6% and 118.7% of the therapeutic effects observed with full MSC‐EVs treatment, respectively. OCT analysis revealed a significant increase in retinal thickness in miR‐486‐3p agomir‐treated mice (Figure 5F,G), with total retinal thickness increasing from 180.4 ± 24.4 µm in scramble‐treated controls to 202.6 ± 19.1 µm following miR‐486‐3p agomir treatment. Furthermore, TEM imaging confirmed that miR‐486‐3p agomir effectively rescued the disrupted RPE/retinal ultrastructure and aberrant mitochondrial morphology characteristic of the NaIO_3_ model (Figure 5H). Preservation of mitochondrial integrity was accompanied by reduced RPE vacuolization and improved cellular organization (Figure 5H–J).

These results demonstrate that miR‐486‐3p is sufficient to replicate the key therapeutic benefits of hucMSC‐EVs in this AMD‐like model. Taken together, our findings strongly support a model where hucMSC‐EVs deliver functional miR‐486‐3p to retinal cells, which then suppresses *Lcn2* expression through direct 3′UTR binding, leading to the inhibition of ferroptosis and consequent preservation of retinal structure and visual function.

## 3 Discussion

This study provides compelling evidence for the potential therapeutic efficacy of MSC‐EVs in the NaIO_3_‐induced AMD‐like model, uncovering a novel molecular mechanism centered on the regulation of ferroptosis via the MSC‐EVs‐miR‐486‐3p/Lcn2 axis. Our findings demonstrate that hucMSC‐EVs, delivered either topically or intravitreally, effectively mitigate retinal degeneration and restore visual function through a well‐defined molecular pathway.

### 3.1 Mechanistic Insights and Therapeutic Innovation

Our mechanistic investigation reveals that the potential protective role of MSC‐EVs is mediated, at least in part, by delivery of miR‐486‐3p, which suppresses the expression of *Lcn2*, a pro‐ferroptotic protein implicated in AMD pathogenesis. This suppression leads to the inhibition of the ferroptosis cascade in RPE and retinal cells, ultimately preserving retinal structure and function. The delineation of the miR‐486‐3p/Lcn2 regulatory axis elucidates a promising molecular target for therapeutic intervention, thereby reinforcing the mechanistic rationale for MSC‐EVs‐based therapies in AMD.

Our finding offers several advantages over existing strategies: (1) it targets a specific molecular pathway rather than providing broad anti‐inflammatory effects; (2) it utilizes the body's natural cargo delivery system (exosomes). While large‐scale production and purification of MSC‐EVs remain challenging, their superior biocompatibility and inherent tissue‐targeting properties may provide important advantages over the synthetic nanocarriers reported in previous studies [26, 27]. (3) it offers multiple administration routes for patient convenience; and (4) it provides a potential mechanism of action for regulatory approval and clinical development. The dual administration routes (intravitreal injection and topical eye drops) both proved effective, with intravitreal injection showing slightly superior efficacy, likely due to direct posterior segment delivery and higher bioavailability.

### 3.2 Clinical Translation and Therapeutic Development

MSC‐EVs represent a promising therapeutic approach for dry AMD. Several factors suggest the potential translational value of MSC‐EVs: (1) MSC‐EVs have demonstrated safety in multiple clinical trials for other indications, with no serious adverse events reported in over 500 patients [28, 29, 30]; (2) the topical administration route offers significant advantages in terms of patient compliance and safety, particularly for chronic conditions requiring long‐term treatment [31]; (3) the identified therapeutic mechanism (miR‐486‐3p/Lcn2 suppression) provides a clear biomarker for treatment response and patient stratification; and (4) the natural origin of EVs reduces immunogenicity concerns compared with synthetic nanoparticles.

However, several challenges must be addressed for successful clinical translation. First, large‐scale production of clinical‐grade MSC‐EVs requires optimization of culture conditions, purification protocols, and quality control measures [32]. Second, the optimal dosing regimen and frequency of administration need to be determined in clinical trials, with consideration for the chronic nature of AMD and the need for sustained therapeutic effects. In support of this consideration, our study demonstrated that undiluted MSC‐EVs exhibited greater efficacy in restoring retinal function and suppressing *Lcn2* expression than diluted preparations (Figure 2I), suggesting that sufficient EV exposure may be required to achieve maximal therapeutic benefit. Further studies are needed to establish a complete dose–response profile and define the optimal therapeutic window. Third, long‐term safety and efficacy data are needed, particularly regarding potential immunogenicity and off‐target effects [33].

### 3.3 Ocular Delivery and Retinal Distribution of hucMSC‐EVs

Our in vivo tracking experiments showed that PKH26‐labeled hucMSC‐EVs were detectable in the retina after both topical instillation and intravitreal injection (Figure S2), providing direct biodistribution evidence for retinal delivery. Following topical administration, MSC‐EVs signals were absent at baseline, became detectable by 3 h, peaked at 12 h, and declined by 24 h in retinal flatmount. Cryosection analysis further revealed penetration into the ganglion cell layer (GCL) and inner nuclear layer (INL) (Figure S2), indicating that topically applied EVs may traverse anterior ocular barriers and reach posterior retinal structures. This pattern is consistent with reports that nanosized vesicles may access the posterior segment via conjunctival–scleral, periocular, or related noncorneal routes that partially bypass the intact corneal epithelial barrier [34, 35, 36], although the precise anterior‐to‐posterior trafficking pathway was not dissected in the present study.

After intravitreal injection, EVs exhibited rapid and robust retinal accumulation within 1 day, with sustained signal at day 3 and progressive clearance by day 6 (Figure S2). Cross‐sectional imaging showed predominant deposition along the GCL, with extension into the INL and RPE‐choroid complex, consistent with diffusion through the vitreous and subsequent interaction with inner layers. Compared with topical delivery, intravitreal administration achieved earlier and denser retinal EV deposition and longer tissue retention on the timescale examined, supporting route‐dependent differences in ocular pharmacokinetics.

Beyond tissue distribution, EVs are internalized by ocular and retinal cells through multiple pathways reported in the literature, including receptor‐mediated endocytosis, macropinocytosis, membrane fusion, and caveolin‐ or clathrin‐dependent uptake [37]. Previous studies have demonstrated efficient EV uptake by retinal neurons, astrocytes, and microglia [38]. Together with our biodistribution data, these findings support the feasibility of using hucMSC‐EVs as natural nanocarriers for retinal cargo delivery, including miR‐486‐3p. We acknowledge that PKH26 labeling primarily reports EVs membrane‐associated fluorescence and may not fully recapitulate the subcellular fate of nonmembrane cargo. Nevertheless, the retinal localization observed here provides a plausible anatomical basis for the functional effects documented in our model and suggests that noninvasive topical EVs administration may offer a patient‐friendly delivery strategy for posterior segment diseases such as AMD, pending further optimization of dose, formulation, and deeper‐layer targeting.

### 3.4 Mechanistic Validation and Therapeutic Targets

Our study reinforces the emerging role of Lcn2 as a critical player in retinal diseases. Elevated Lcn2 has been associated not only with AMD but also with glaucoma, where its neutralization showed therapeutic benefits [22, 23]. The recent link between Lcn2 and RPE ferroptosis [24] aligns perfectly with our findings that MSC‐EVs treatment suppresses Lcn2 while inhibiting ferroptosis markers. By demonstrating that *Lcn2* overexpression phenocopies AMD‐like pathology and that MSC‐EVs can rescue this phenotype, we consider that Lcn2 is a high‐value target for dry AMD.

The identification of miR‐486‐3p as the key factor in MSC‐EVs is particularly significant. miR‐486‐3p is a well‐conserved microRNA with established roles in various biological processes, including cell survival, oxidative stress response, and iron homeostasis. Notably, the finding that the miR‐486‐3p agomir mirrored the functional rescue conferred by intact MSC‐EVs highlights this microRNA as a primary therapeutic effector. Consequently, miR‐486‐3p represents a promising drug candidate that could be harnessed via bioengineered EVs or synthetic nanocarrier platforms. The conservation of miR‐486‐3p across mammalian species (100% identity between human and mouse) supports its translational potential.

### 3.5 Ferroptosis as a Therapeutic Target

Ferroptosis is increasingly recognized as a central mechanism in AMD pathogenesis, and our study adds to the growing body of evidence supporting ferroptosis inhibition as a therapeutic strategy. While pathways like Nrf2 signaling are known regulators of ferroptosis [39], our identification of the MSC‐EVs miR‐486‐3p/Lcn2 axis adds another layer to this complex regulatory network. This finding provides a novel strategy to specifically counteract ferroptosis in RPE cells using a biologically derived therapeutic agent.

The coordinated regulation of multiple ferroptosis markers (Lcn2 downregulation, Gpx4 upregulation, GSH restoration, ferritin downregulation) suggests that MSC‐EVs target the ferroptosis pathway at multiple levels, providing robust protection against oxidative damage. This multi‐target approach may be more effective than single‐target interventions, particularly given the complex and redundant nature of oxidative stress responses in the retina.

### 3.6 Study Limitations and Future Directions

Several limitations of this study should be acknowledged. First, the NaIO_3_ model primarily induces acute RPE toxicity rather than recapitulating all chronic features of human dry AMD, such as drusen formation or slow progression of geographic atrophy. Future studies should validate these findings in other preclinical models, potentially those involving chronic oxidative stress (e.g., ApoE4 models) or genetic predisposition (e.g., *Ccl2* ^−/−^ or *Cx3cr*1^−/−^ models).

Second, MSC‐EVs naturally contain a highly complex dynamic cargo, including diverse microRNAs, proteins, and lipids. Although we confirmed the direct binding between miR‐486‐3p and Lcn2 as well as the rescue effect of the miR‐486‐3p agomir, we cannot completely rule out the possibility that other bioactive constituents within MSC‐EVs act synergistically to confer protection in AMD models. While the gain‐of‐function experiments in this study established the sufficiency of miR‐486‐3p, rigorous loss‐of‐function approaches such as utilizing miR‐486‐3p‐depleted EVs or Lcn2‐overexpressing rescue vectors in vivo are still required to definitively prove its absolute necessity. Future studies using conditional knockout models or stable miRNA‐inhibited EV delivery systems will be essential to precisely quantify the specific contribution ratio of EVs‐derived miR‐486‐3p in retinal protection and to fully elucidate the underlying cooperative network.

Third, the long‐term stability and persistence of therapeutic effects need to be investigated, particularly for the topical administration route. Studies examining the pharmacokinetics and biodistribution of MSC‐EVs in the eye would provide valuable insights for clinical translation. Preliminary data suggest that EVs can persist in the retina for up to 7 days postadministration, but longer‐term studies are needed.

### 3.7 Future Directions and Clinical Development

Several promising directions emerge from this work. First, compared with engineered EV platforms that rely on cargo loading or surface modification to enhance targeting efficiency, native hucMSC‐EVs offer inherent advantages in manufacturing simplicity, biocompatibility, and translational feasibility [40]. However, their relatively limited targeting specificity and cargo‐loading capacity may constrain therapeutic efficacy [40]. Given the central role of miR‐486‐3p in mediating retinal protection, future engineering approaches aimed at enhancing retinal and RPE‐cell tropism or enriching miR‐486‐3p cargo may further improve the efficacy of EVs‐based therapies for dry AMD [41]. Potential strategies include genetic modification of parental MSCs, EVs surface engineering, and hybrid delivery systems that combine the biological advantages of EVs with the tunable properties of synthetic nanomaterials. Second, the identification of additional therapeutic cargo within MSC‐EVs could lead to combination therapies with synergistic effects. Preliminary analysis suggests that MSC‐EVs contain multiple miRNAs with anti‐inflammatory and antioxidative properties, including miR‐22, miR‐146a, and miR‐124, which may work synergistically with miR‐486‐3p [42, 43, 44]. Third, the development of synthetic delivery systems for miR‐486‐3p could provide alternatives to EV‐based delivery. Current approaches include lipid nanoparticles, polymeric nanoparticles, and cell‐penetrating peptides, each with advantages and limitations for ocular delivery.

### 3.8 Clinical Implications and Regulatory Considerations

The findings from this study have significant clinical implications for dry AMD treatment. The identification of a specific therapeutic mechanism (miR‐486‐3p/Lcn2 suppression) provides a clear target for drug development and a potential biomarker for treatment response. The demonstration of efficacy through both invasive and noninvasive delivery routes offers flexibility in treatment approaches, potentially allowing for personalized therapy based on disease severity and patient preference.

For regulatory approval, the natural origin of MSC‐EVs may provide advantages over synthetic nanoparticles, but rigorous quality control and batch‐to‐batch consistency will be essential. The FDA has recently issued guidance on the development of cell and gene therapy products, which may be applicable to EVs‐based therapies. European regulatory agencies have also established guidelines for advanced therapy medicinal products (ATMPs) that may facilitate EVs therapy development.

### 3.9 Conclusion

In conclusion, our study demonstrates that MSC‐EVs represent a good therapeutic option for the NaIO_3_‐induced AMD‐like model. We elucidate a novel mechanism whereby MSC‐EVs delivery of miR‐486‐3p suppresses *Lcn2* expression, inhibits ferroptosis in retinal cells, and significantly rescues retinal electrophysiological responses and preserves retinal morphology. These findings provide a strong rationale for further development of MSC‐EVs‐based therapies targeting the miR‐486‐3p/Lcn2/ferroptosis axis for dry AMD

## 4 Materials and Methods

### 4.1 Animals

C57BL/6 mice which regardless of sex, were provided by the Institute of Laboratory Animals of Sichuan Academy of Medical Sciences & Sichuan Provincial People's Hospital and were raised in a specific pathogen‐free room with a 12 h:12 h light cycle in the institute. AMD‐like mouse model was generated by NaIO_3_ administration (15 mg/kg, i.p.) according to the 2‐month‐old mice's weight. After 2 weeks of modeling, the AMD‐like mice received hucMSC‐EVs treatment. All animal work in this study was approved by the Ethical Committee of Sichuan Academy of Medical Sciences & Sichuan Provincial People's Hospital (approval no. 2026–478). Animal procedures were performed in compliance with the ARVO Statement on the Use of Animals in Ophthalmic and Vision Research and followed the institutional animal care guidelines. At least three individuals were used for each experiment.

### 4.2 hucMSC‐EVs Preparation

hucMSC‐EVs were purchased from HUAMEI BIOTECH company using 3D culture technology to enhance exosome yield and biological activity [45, 46, 47]. Briefly, fresh umbilical cords were harvested from full‐term, cesarean‐section puerperae without any diseases under Ethics Committee approval. Subsequently, MSC‐EVs were isolated according to established protocols, and their safety profile was thoroughly evaluated [45, 46, 47]. The morphology and size distribution of MSC‐EVs were characterized by TEM and NTA, respectively. Protein concentration was determined using a BCA protein assay kit (Beyotime, #P0010S), and the particle‐to‐protein ratio was calculated as an additional quality control parameter. EVs identity was further confirmed by western blot analysis of canonical EVs markers, including CD9 (ABclonal, #A19027), CD63 (Proteintech, 67605‐1‐Ig), and TSG101 (ABclonal, A5789), while the endoplasmic reticulum marker Calnexin was used as a negative control. For in vivo tracking, hucMSC‐EVs were labeled with dye PKH26 (Beyotime, #C3637S) following the manufacturer's protocol. Briefly, 10 µg of MSC‐EVs were mixed with 100 µL PKH26 labeling solution and incubated for 5 min at room temperature in the dark. The labeling reaction was terminated by adding an equal volume of stop solution. PKH26‐labeled EVs were subsequently used for retina and RPE uptake assays. PKH26‐positive puncta were quantified within each imaging field.

### 4.3 Intravitreal Injection

Mice were anesthetized with tribromoethanol (200 mg/kg) and dilated pupils with 1% topiramine eye drops. Small puncture was made using 30 g needle (Becton, Dickinson and company, 30 g × 1/2) at the lower edge of the mouse eye equator. 2 µL MSC‐EVs, adeno‐associated viruses, or microRNAs containing 0.5% sodium fluorescein were injected into the vitreous using a microsyringe (syringe, Hamilton No. 7632‐01; Needle, Hamilton No. 7803–05) and then waiting for 10 s until the needle was withdrawn from vitreous. Control group mice were injected with physiological saline (pH 7.4) using the same method.

### 4.4 Eyeball Cryosection Slides Preparation and Staining

The intact eyeballs were dissected and immediately fixed in 4% paraformaldehyde (PFA) for 10 min at room temperature. A small puncture was rapidly made at the center of each eyeball to facilitate fixative penetration, followed by continued fixation on ice for 2 h. After fixation, samples were washed three times with PBS (pH 7.4), each time lasting 5 min. Subsequently, tissues were dehydrated in 30% sucrose solution at 4°C for 12 h. Under a dissecting microscope, the cornea was excised along the equator, the lens was carefully removed with forceps, and residual fluid was aspirated. The processed eyeballs were then immersed in embedding medium (SAKURA, Tissue‐Tek O.C.T. Compound, #4583), snap‐frozen at −80°C for 30 min or stored at −80°C until sectioning. 12 µm‐thick cryosections were prepared using a cryostat microtome. For immunostaining, retinal cryosection slides were blocked with 3% bovine serum albumin (BSA; #A9647, Sigma‐Aldrich) and 0.1% Triton X‐100 in PBS (PBST) for 2 h at room temperature. The sections were then incubated overnight at 4°C with a primary antibody against RPE65 (ABclonal, #A9615) at the recommended dilution. After washing three times with PBST, slides were incubated with a fluorophore‐conjugated anti‐rabbit secondary antibody and DAPI for 2 h at room temperature. Imaging was performed using a confocal laser scanning microscope (Zeiss LSM 900).

### 4.5 Retinal and RPE Flatmount Preparation and Staining

Eyes were immediately enucleated and fixed in 4% PFA in PBS at room temperature for 1 h. The anterior segment, including the cornea, lens, and vitreous body, was carefully removed to yield an eye cup. The retina and RPE layer were gently separated using fine forceps.

To facilitate flat mounting, four radial incisions extending from the periphery toward the optic nerve head were made to divide the retina or RPE into a cloverleaf shape. The dissected retinas or RPE layers were washed three times with PBS (5 min each), blocked with 3% bovine serum albumin and 0.1% PBST at room temperature for 2 h. To delineate RPE cell borders, the tissues were incubated overnight at 4°C with primary antibodies targeting RPE junctional anti‐ZO‐1 (ABclonal, #A28491). After washing three times with PBST, slides were incubated with a fluorophore‐conjugated anti‐rabbit secondary antibody and DAPI for 2 h at room temperature. Then, the retinas or RPE layer were flat‐mounted on glass slides with the ganglion cell layer (GCL) facing upwards, coverslipped using an anti‐fade mounting medium, and imaged using a confocal laser scanning microscope (Zeiss LSM 900).

### 4.6 Hematoxylin–Eosin Staining

Mouse eyes were fixed using 4% PFA at room temperature overnight. The samples were immersed in dewaxing reagent (Servicebio, #G1128) for 20 min after sectioning, followed by rehydration by dipping 5 min in each of 100%, 95%, and 75% ethanol. After being removed from the −20°C freezer, the frozen slides were equilibrated to room temperature and subsequently fixed with tissue fixative (Servicebio, #G1101) for 15 min. Slides were incubated in hematoxylin solution for 5 min, followed by rinsing in water for 10 min, being dehydrated in 95% ethanol for 1 min, and subsequently stained in eosin solution for 15 s. Finally, the slides were dehydrated again and immersed in xylene to keep them transparent, followed by mounting with neutral balsam. Slides were analyzed under a light microscope (Nikon Eclipse E100, Japan).

### 4.7 Electroretinogram

The experimental mice were dark‐adapted for 12 h in a light‐tight chamber. All subsequent procedures, including anesthesia and pupil dilation, were performed under dim red light to maintain dark adaptation. The mice were positioned on the ERG (Diagnosys, Celeris) constant‐temperature (37°C) heated platform, and the electrode was recorded in contact with the central cornea of the mouse by the setting program. The reference electrode and ground needle electrode were connected and operated according to the instructions of the detector. ERG data of both eyes were recorded and processed.

### 4.8 Optical Coherence Tomography

Following anesthesia and pupil dilation, the OCT (MICRON Image‐Guided OCT2 system, Phoenix‐Micron, USA) lens was placed directly in front of the mouse eye. Guided by real‐time images on the OCT system display, the lens was adjusted to ensure proper contact with the ocular surface. The three‐dimensional stage was maneuvered until a clear fundus image was visualized on the monitor. Subsequently, the clarity of retinal tomographic images was optimized. All resultant images were captured and archived for subsequent analysis.

### 4.9 Optomotor Response Assays

Optomotor response (OMR) reaction is usually used for eye function evaluation (OptoTrack XR‐OT101 system, OptoTrack Version 4, XINRUN). For this part, we used a stable‐frequency (0.2 cyc/deg) wave to stimulate the mice and auto‐record the time and right rate for the mice according to their head‐waving direction. After randomly changing the direction of the wave several times, the Visual reflex index was calculated. Three mice from different groups were tested.

### 4.10 Transmission Electron Microscope

After being euthanized, mice's eyeballs were immediately removed to 10% neutral PFA for fixation for at least 24 h. Each group of eyeballs was sectioned into 1 mm × 2 mm pieces, rapidly immersed in 2.5% glutaraldehyde, and then fixed in 1% osmium tetroxide (OsO_4_). The samples underwent dehydration using a graded ethanol series ranging from 30% to 100%, followed by embedding in epoxy resin. Ultrathin sections were prepared with an ultramicrotome, then placed on copper grids that had been coated with a carbon film. These grids were examined using a Philips CM120 TEM (CM120, Philips, the Netherlands).

### 4.11 Production of Recombinant AAV2 Viral Vectors

For *Lcn2* overexpression, the pAAV‐CMV‐Lcn2‐P2A‐GFP plasmid was constructed using the *CMV* promoter to initiate gene expression, a Kozak sequence GCCACC in front of the target gene's ATG was added, and a flag tag at the C‐terminus of the target gene was added to express *Lcn2* and *GFP* through the P2A nonfusion method. AAV2 adeno‐associated virus expressing only GFP was used as a control. *Lcn2*‐overexpression plasmid was co‐transfected with the packaging plasmid into HEK‐293T cells. After 48 h, the packaged virus was collected from the culture medium and filtered with a 0.22 µm SYRINGE FILTER (Biosharp, #BS‐PES‐22) for subsequent virus concentration. The final concentration of the virus was 1 × 10^13^ viral genomes/mL, and the virus was stored in a balanced salt solution (BSS) (Alcon, Fort Worth, Texas) with 0.014% Tween‐20.

### 4.12 Western Blot

Total proteins from mouse retinal tissues were extracted in PBS and lysed using RIPA buffer (Solarbio, #R0020) containing 1 mM PMSF (Solarbio, #P0100). After extraction, the supernatant was collected to quantify the protein concentration using Enhanced BCA Protein Assay Kit (Beyotime, #P0010S). Next, SDS‐PAGE electrophoresis was performed by loading equal amounts of protein in the samples containing SDS‐PAGE sample loading buffer (Beyotime, #P0015) on 15% SDS‐PAGE gels. All proteins were transferred to 0.45 µm nitrocellulose membrane (BioRad, #1620145). Then, the membranes were blocked with BSA (Beyotime, #P0231) at room temperature for 1 h. Primary antibodies were used to incubate membranes at 4°C overnight. Anti‐Lcn2 (1:500, #AF7362), anti‐Ferritin (1:500, #AF2104), and anti‐Gpx4 (1:800, #AF7020) were purchased from Beyotime, and anti‐β‐actin was purchased from ABclonal (1:5000, #AC038). The following second antibody, HRP‐conjugated Goat anti‐Rabbit IgG (H+L) (1:10,000, ABclonal, #AS014), was used to incubate membranes for 1 h at room temperature.

### 4.13 RNA Extraction and RT‐qPCR

Six retina tissues per group were dissected in PBS and collected to extract total RNA using the RNAsimple total RNA kit (Tiangen Biotech, #DP419). 1 µg total RNA was used to synthesize cDNA according to the protocol of ABScript Neo RT Master Mix for qPCR with gDNA Remover (ABclonal, #RK20433). RT‐qPCR was performed with a 2X Universal SYBR Green Fast qPCR Mix (ABclonal, #RK21203) using a 7500 fast real‐time PCR System (Applied Biosystems, Carlsbad, CA, USA). *Actin* was used as the internal control gene. All primers used for RT‐qPCR are listed in Table S3. All experiments were independently performed with three biological replicates, and the relative mRNA expression levels were calculated using the 2^−ΔΔC^ *^T^* method.

### 4.14 RNA‐seq Analysis

After treating the NaIO_3_‐induced model with MSC‐EVs, mice were euthanized, and their retinas were dissected to extract total RNA and further processed for RNA‐seq using Illumina Novaseq 6000. The raw reads were processed with the BMKCloud (www.biocloud.net) online platform and submitted to the National Genomics Data Center, China (https://ngdc.cncb.ac.cn), with the project number PRJCA069778. Clean data (clean reads) were obtained by removing reads containing adapters, reads containing ploy‐N, and low‐quality reads from raw data. All the downstream analyses were based on clean data of high quality. Clean data were mapped to the mouse reference genome downloaded from UCSC (https://hgdownload.soe.ucsc.edu/downloads.html#mouse) accurately using HISAT2 (v2.0.4) software. Differential expression analysis was performed using DESeq2 (1.30.1). Genes with an adjusted *p*‐value < 0.01 and a fold change of ≥2 were assigned as significantly differentially expressed. Gene function was annotated based on the following databases: KO (KEGG Ortholog database) and GO (Gene Ontology).

### 4.15 Measurement of Glutathione Level

For total glutathione (GSH) measurement, we used a commercial GSH detection kit (Beyotime, #S0053). According to the instructions of this kit, we tested the total GSH level after EV treatment. In brief, we harvested three retina tissues from mice in different groups and mixed them into one sample. We made three duplicate wells and added the reaction reagent to the sample well. After the chemical reaction, the absorbance wave at around 412 nm was detected by the Microplate Reader. After characterizing the standard curve by the GSH reference from the kit, the concentrations of different samples were detected. The data were calculated, and statistical analysis was performed.

### 4.16 Dual‐Luciferase Reporter Assay

To validate the interaction between miR‐486‐3p and the 3′UTR of *Lcn2*, the WT *Lcn2* 3′UTR fragment containing the predicted miR‐486‐3p binding site and the corresponding MUT fragment were synthesized and cloned into the pmirGLO dual‐luciferase reporter vector. HEK‐293T cells were co‐transfected with either WT or MUT reporter constructs and miR‐486‐3p mimics or negative control (NC) mimics using HighGene Transfection Reagent (ABclonal, #RM01091). For each well of a 24‐well plate, 0.2 µg of reporter plasmid and 50 nM miRNA mimics were used. After 24 h of transfection, luciferase activities were measured using a dual‐luciferase reporter assay kit (Yeasen Biotechnology, #11402ES60) according to the manufacturer's instructions. Firefly luciferase activity was normalized to Renilla luciferase activity, and the relative luciferase activity was calculated for subsequent analysis.

### 4.17 ROS Reporter Assay

ROS levels in freshly isolated mouse retinal tissues were quantified using a DCFH‐DA fluorescent ROS detection kit (Beyotime, #S0033S). Tissues were homogenized in 300 µL of NP‐40 lysis buffer (Beyotime, #P0013F) and centrifuged at 12,000 rpm for 10 min at 4°C, and the collected supernatants were used for subsequent detection. Each 60 µL aliquot of tissue lysate was diluted with 250 µL PBS, supplemented with DCFH‐DA working solution at a 1:1000 dilution, and fully mixed. A 100 µL portion of the mixture was transferred to a black 96‐well plate and incubated at 37°C for 30 min in the dark. DCF fluorescence intensity was measured by a microplate reader at excitation and emission wavelengths of 488 and 525 nm, respectively. Total protein content of lysates was quantified via a BCA protein assay kit, and relative ROS levels were determined by normalizing fluorescence intensity to corresponding total protein concentrations.

### 4.18 Statistical Analysis

All statistical analyses were performed using GraphPad Prism 10.1.2 software. Comparisons between two independent groups were performed using an unpaired two‐tailed Student's *t*‐test. For comparisons among multiple groups, one‐way analysis of variance (ANOVA) followed by Dunnett's or Tukey's post hoc test, as appropriate, was used. Quantitative data are presented as mean ± standard deviation (SD). Statistical significance was defined as *p *< 0.05, and is indicated as follows: * *p < *0.05, ** *p < *0.01, and *** *p < *0.001.

## Author Contributions

L.H. designed this study. Z.L., T.Z., L.Y., H.T., and R.L. performed the experiments. The original draft was written by Z.L. and T.Z. L.H. reviewed and edited the manuscript. All authors have read and approved the final manuscript.

## Ethics Statement

This study was approved by the Ethical Committee of Sichuan Academy of Medical Sciences & Sichuan Provincial People's Hospital (Approval No. 2023 –478). Animal procedures were performed in compliance with the ARVO Statement on the Use of Animals in Ophthalmic and Vision Research and followed the institutional animal care guidelines.

## Conflicts of Interest

The authors have filed a patent application related to the findings presented in this manuscript with the China National Intellectual Property Administration (patent application no. 202610997023.7). The remaining authors declare no conflicts of interest.

## Supporting information




**Supporting Information file**: mco271000‐sup‐0001‐SuppMat.zip

## Data Availability

The raw RNA sequencing data from NaIO_3_‐induced AMD‐like mice treated with MSC‐EVs (via intravitreal injection or eye drops) or saline have been uploaded to the Genome Sequence Archive (Genomics, Proteomics & Bioinformatics 2025) in the National Genomics Data Center (Nucleic Acids Res 2026), China National Center for Bioinformation/Beijing Institute of Genomics, Chinese Academy of Sciences (https://ngdc.cncb.ac.cn). The project accession number is PRJCA069778.
