# Supplementary material for: Mesenchymal Stem Cell‐Derived Extracellular Vesicles Ameliorate NaIO3‐Induced Dry AMD by Delivering miR‐486‐3p to Inhibit Lipocalin‐2‐Mediated Ferroptosis
Source: MedComm (2020). 2026 Sep 16;7(10):e71000. doi: 10.1002/mco2.71000 (PMC13583448; doi:10.1002/mco2.71000)
Supplement: Supplementary file 1 [file MCO2-7-e71000-s001.zip › Supplementary Information.docx]

**Mesenchymal Stem Cell-Derived** **Extracellular Vesicles Ameliorate NaIO_3_-induced Dry AMD by Delivering miR-486-3p to Inhibit Lipocalin-2-Mediated Ferroptosis**

Zheng Li^1#^, Tujing Zhao^1#^, Lin Ye^1^, Runze Li^1^, Huaping Tian^1^, Ruilin Liao^1^, and Lulin Huang^1，2*^

^1^The Genetic Diseases Key Laboratory of Sichuan Province, The Department of Medical Genetics, The Department of Laboratory Medicine, Sichuan Academy of Medical Sciences & Sichuan Provincial People's Hospital, School of Medicine, University of Electronic Science and Technology of China, Chengdu, China.

^2^Research Unit for Blindness Prevention, Chinese Academy of Medical Sciences (2019RU026), Sichuan Academy of Medical Sciences and Sichuan Provincial People’s Hospital, Chengdu, Sichuan, China.

 *Corresponding authors. Email: [huangluling@yeah.net](mailto:huangluling@yeah.net) (Lulin Huang).

^#^These authors contributed equally to this work.

**FIGURE S1 Characterization of MSC-derived Extracellular Vesicles.** (A) Nanoparticle tracking analysis (NTA) profile displaying the size distribution and concentration of diluted MSC-EVs. (B) Representative TEM image displaying typical cup-shaped MSC-EVs morphology. Scale bar = 100 nm. (C) Western blot analysis confirming enrichment of exosome markers (CD9, CD63, TSG101) in purified MSC-EVs lysates compared to source mesenchymal stem cell lysates. The absence of the endoplasmic reticulum marker Calnexin in the MSC-EVs fraction indicates purity from cellular contamination.

**FIGURE S2 Distribution and Localization of PKH26-labeled EVs in The Retina and RPE.**

(A) PKH26-labeled EVs (PKH26-EV; red) at 3, 6, 12, and 24 h after eye-drop administration. (B) Quantification of PKH26-positive puncta per field in (A). n = 3. (C) PKH26-EVs at 1, 3, and 6 days after intravitreal injection. (D) Quantification of PKH26-positive puncta per field in (C). n = 3. (E) PKH26 fluorescence and quantification at 12 h after eye-drop administration of PKH26 diluted in PBS (PKH26-PBS) or PKH26-EV. n = 4. (F) PKH26 fluorescence and quantification at 1 day after intravitreal injection of PKH26-PBS or PKH26-EVs. n = 3. (G, H) Retinal cryosections showing PKH26-EVs distribution at 3, 6, 12, and 24 h after eye-drop administration (G) and at 1, 3, and 6 days after intravitreal injection (H). (I) Retinal cryosections at 3 days after intravitreal injection of PKH26-PBS or PKH26-EVs. PKH26, red; RPE65, green; DAPI, blue. (J) RPE flat mounts and quantification at 12 h after eye-drop administration of PKH26-PBS or PKH26-EVs. PKH26, red; ZO-1, green. n = 3. (K) RPE flat mounts and quantification at 6 days after intravitreal injection of PKH26-PBS or PKH26-EVs. PKH26, red; ZO-1, green. n = 4. ONL, outer nuclear layer; INL, inner nuclear layer; GCL, ganglion cell layer; RPE, retinal pigment epithelium. Scale bars, 20 μm (A, C), 50 μm (E–K). Data are presented as mean ± SD. *P < 0.05; NS, not significant.

**FIGURE S3 Differentially Expressed Genes and Enriched Pathways in Intravitreal Injection Group.** (A) Heatmap showing DEGs in NaIO_3_-induced AMD-like model mice treated with MSC-EVs via intravitreal injection. (B) GO enrichment analysis highlighting biological processes such as ion transport and cell adhesion. (C) KEGG pathway analysis revealing involvement of PI3K-Akt signaling and complement cascades.

**FIGURE S4 Differentially Expressed Genes and Enriched Pathways in Eye Drop Group.** (A) Volcano plot showing DEGs in NaIO_3_-induced AMD-like model mice treated with MSC-EVs via eye drops. (B) Heatmap showing the expression profiles of differentially expressed genes in the eye drops group compared with controls (C) GO enrichment analysis highlighting biological processes related to immune response and positive regulation of protein phosphorylation. (D) KEGG pathway analysis revealing pathways such as proteasome and viral infection.

**Table S1 All Expressed miRNA in MSC-EVs Identified in This Study**

**Table S2 The WT and MUT *Lcn2* 3**′**UTR sequences**

WT-Lcn2-UTR

Sense:5’ATCAAAAAAGGCCCAGGCAGCCGAGATCACCCACATGTCTCCCTCTGCTCTGTTCTGATGAGCTTTCTCTCCTACAGACCAATGCATTGACAACTGAATGGGTGGTGAGTGTGGCTGACTGGGATGCGCAGAGACCCAATGGTTCAGGCGCTGCCTGTCTGTCTGCCACTCCATCTTTCCTGTTGCCAGAGAGCCACCTGGCTGCCCCACCAGCCACCATACCAAGGAGCATCTGGAGCCTCTTCTTATTTGGCCAGCACTCCCCATCCACCTGTCTTAACACCACCAATGGCGTCCCCTTTCTGCTGAATAAATACATGCCCCCACTTCTCAGGGTCTGTGCTACTTAATCCCATAACAATACCCATAACAATGAAGGGCAAAAGAGCAAAGGGAGGCTGGAGAGAT3’

MUT-Lcn2-UTR

Sense:5’ATCAAAAAAGGCCCAGGCAGCCGAGATCACCCACATGTCTCCCTCTGCTCTGTTCTGATGAGCTTTCTCTCCTACAGACCAATGCATTGACAACTGAATGGGTGGTGAGTGTGGCTGACTGGGATGCGCAGAGACCCAATGGTTCAGGCGCTGCCTGTCTGTCTGCCACTCCATCTTTCCTGTTGCCAGAGAGCCACCTGGAGGAAAAACCAGCCACCATACCAAGGAGCATCTGGAGCCTCTTCTTATTTGGCCAGCACTCCCCATCCACCTGTCTTAACACCACCAATGGCGTCCCCTTTCTGCTGAATAAATACATGCCCCCACTTCTCAGGGTCTGTGCTACTTAATCCCATAACAATACCCATAACAATGAAGGGCAAAAGAGCAAAGGGAGGCTGGAGAGAT3’

miR-486-3p-mimics

Sense: 5’CGGGGCAGCUCAGUACAGGAU3’

Antisense: 5’CCUGUACUGAGCUGCCCCGuu3’

miR-486-3p-mimics NC :

Sense: 5’UUGUACUACACAAAAGUACUG3’

Antisense: 5’GUACUUUUGUGUAGUACAAUU3’

miR-486-3p-agomir

Sense:5’CGGGGCAGCUCAGUACAGGAU3’

Antisense:5’(mC)*(mC)*(mU)(mG)(mU)(mA)(mC)(mU)(mG)(mA)(mG)(mC)(mU)(mG)(mC)(mC)(mC)*(mC)*(mG)(mU)(mU)3’

miR-486-3p-agomir NC

Sense:5’UCACAACCUCCUAGAAAGAGUAGA3’

Antisense:(mU)*(mC)*(mU)(mA)(mC)(mU)(mC)(mU)(mU)(mU)mC)(mU)(mA)(mG)(mG)(mA)(mG)(mG)(mU)(mU)(mG)*(mU)*(mG)*(mA)3’

**Table S3 All RT-qPCR primers used in This Study**

| *Actb-F* | GGCTGTATTCCCCTCCATCG |
| --- | --- |
| *Actb-R* | CCAGTTGGTAACAATGCCATGT |
|  |  |
| *Steap4-F* | GGGAAGTCACTGGGATTGAAAA |
| *Steap4-R* | CCGAATAGCTCAGGACCTCTG |
|  |  |
| *Ftl1-F* | CCATCTGACCAACCTCCGC |
| *Ftl1-R* | CGCTCAAAGAGATACTCGCC |
|  |  |
| *Serpina3n-F* | CCGGAGGCCCTTTACTACCA |
| *Serpina3n-R* | GGAGTAGGGGTGAGTAGGCAG |
|  |  |
| *Gfap-F* | CCCTGGCTCGTGTGGATTT |
| *Gfap-R* | GACCGATACCACTCCTCTGTC |
|  |  |
| *Lcn2-F* | CCACCTCAGACCTGATCCCA |
| *Lcn2-R* | CCCCTGGAATTGGTTGTCCTG |
